# Healthcare Access in Chemsex Contexts in Brazil: A Scoping Review and the VIP-Chemsex Model

**DOI:** 10.3390/nursrep16070238

**Published:** 2026-07-09

**Authors:** Isadora Silva de Carvalho, Lariane Angel Cepas, Álvaro Francisco Lopes de Sousa, Talita Morais Fernandes, Talia Gomes Luz, Ruan Nilton Rodrigues Melo, Mayara Souza Gomes, Caíque Jordan Nunes Ribeiro, Anderson Reis de Sousa, Inês Fronteira, Fátima Morales, Ricardo Nakamura, Ana Paula Morais Fernandes

**Affiliations:** 1 Department of General and Specialized Nursing, Ribeirão Preto School of Nursing, University of São Paulo, Ribeirão Preto 14040-902, Brazil; isadoracarvalho@usp.br (I.S.d.C.); larianeangelcepas@usp.br (L.A.C.); taliagomesluz@usp.br (T.G.L.); ruan.melo@usp.br (R.N.R.M.); mayarasgomes@usp.br (M.S.G.); anapaula@eerp.usp.br (A.P.M.F.); 2 Campus Três Lagoas, Federal University of Mato Grosso do Sul (UFMS), Três Lagoas 79070-900, Brazil; 3 Public Health Research Centre, Comprehensive Health Research Center (CHRC), NOVA University Lisbon, 1099-085 Lisbon, Portugal; ines.fronteira@ensp.unl.pt; 4 Department of Sport Sciences, Higher Institute of Lisbon and Tagus Valley, 2620-379 Lisbon, Portugal; talitafernandes@usp.br; 5 Graduate Program of Nursing, Federal University of Sergipe, São Cristóvão 49107-230, Brazil; caiquejordan@academico.ufs.br; 6 School of Nursing, Federal University of Bahia, Salvador 40170-110, Brazil; anderson.sousa@ufba.br; 7 Department of Preventive Medicine and Public Health, School of Medicine, University of Seville, 41009 Seville, Spain; fmmarin@us.es; 8 Department of Computer Engineering and Digital Systems, Polytechnic School, University of São Paulo, São Paulo 05508-010, Brazil; ricardonakamura@usp.br

**Keywords:** chemsex, sexualized drug use (SDU), sexually transmitted infections (STIs), sexual and gender minorities, harm reduction, health policy, nursing

## Abstract

**Background/Objectives:** Sexualized drug use (SDU) and chemsex have emerged as a growing public health concern globally, reflecting complex intersections between sexual practices, psychoactive substance use, and structural vulnerabilities. In Brazil, however, evidence on healthcare access among individuals who engage in SDU/chemsex remains limited and fragmented. This scoping review aimed to map and analyze the available literature on healthcare access in this population, identifying barriers, facilitators, and gaps in care. **Methods:** The review followed the Arksey and O’Malley framework and Joanna Briggs Institute recommendations, with searches conducted in six databases (MEDLINE/PubMed, Embase, Scopus, SciELO, LILACS, and PsycINFO) for studies published between 2014 and 2025. **Results:** Eleven studies met the inclusion criteria, predominantly quantitative and concentrated in large urban centers. Findings indicate that healthcare access is shaped by persistent structural and symbolic barriers, including stigma, discrimination, fear of disclosure, and limited professional preparedness. Care remains largely centered on human immunodeficiency virus (HIV) and sexually transmitted infections (STIs) services, with insufficient integration of primary care, mental health, and substance use services, contributing to fragmented care. Significant gaps were identified, including the underrepresentation of women, transgender, and non-binary populations, and the absence of studies focusing on healthcare professionals. **Conclusions:** Substance use patterns reflect both global trends and local specificities, particularly the prominence of alcohol and cocaine in Brazil. This review provides the first synthesis of Brazilian evidence on chemsex from a healthcare access perspective. The findings highlight critical inequities and support the need for integrated, stigma-free, and context-sensitive care within the Brazilian Unified Health System. Based on these findings, the VIP-SDU/Chemsex Model is proposed as a multilevel framework to explain how structural, symbolic, and programmatic factors shape access and health outcomes.

## 1. Introduction

The intersection between psychoactive substance use and sexual practices has emerged as a complex and multifaceted phenomenon in the field of public health, particularly among socially vulnerable populations. In this context, the term chemsex, derived from the combination of the words “chemical” and “sex,” has been widely used to describe the intentional use of psychoactive substances before or during sexual encounters, with the aim of intensifying, prolonging, or enhancing sexual experiences. Originally described in urban contexts in the United Kingdom, particularly among gay, bisexual, and other men who have sex with men (MSM), chemsex was initially associated with the use of specific substances, such as methamphetamine, gamma-hydroxybutyrate (GHB/GBL), and mephedrone, often in prolonged sexual sessions involving multiple partners [1,2].

Despite its widespread dissemination in the international literature, the concept of chemsex remains heterogeneous and dependent on the sociocultural context in which it is applied [3]. There is no universal consensus regarding the substances that constitute it or the behavioral criteria that define it, resulting in significant variability in prevalence estimates and methodological approaches adopted in studies [4]. This conceptual ambiguity becomes even more relevant when the term is applied to contexts different from those in which it was originally conceived, especially in low- and middle-income countries [5].

In parallel, the broader concept of sexualized drug use (SDU) has gained prominence in the literature. SDU refers to the use of any psychoactive substance immediately before or during sexual activity, regardless of the type of drug, frequency of use, or the context in which it occurs. Unlike chemsex in its more restrictive definition, SDU encompasses a wide variety of substances, including alcohol, cocaine, cannabis, poppers, ecstasy, and medications for erectile dysfunction, reflecting a broader and more heterogeneous spectrum of practices and motivations [6,7].

Although chemsex can be understood as a subset of SDU, characterized by specific patterns of substance use and particular social dynamics, the boundaries between these concepts are often blurred in empirical practice. In many contexts outside Europe, substance use in sexual settings does not necessarily follow the classic chemsex model described in the United Kingdom but is instead influenced by local factors such as drug availability, cultural norms, socioeconomic conditions, and structural inequalities [8,9].

This distinction becomes especially relevant in the Brazilian context [10]. Brazil presents a unique epidemiological and sociocultural scenario in which substance use in sexual contexts is relatively frequent but does not always fit the traditional chemsex pattern [11]. National evidence indicates that substances such as alcohol, cocaine, cannabis, and medications for sexual performance are widely used in sexual contexts, often in less structured ways and not necessarily linked to organized events or prolonged sessions [12,13].

In this sense, the exclusive adoption of the term chemsex may limit the understanding of the phenomenon in the country, leading to an underestimation of its magnitude and to the invisibility of practices that are relevant to public health. Recent studies [12] have highlighted that, in contexts such as Latin America, the use of the concept of SDU may be more appropriate, as it allows for an approach that is more sensitive to local specificities and to the diversity of substance use patterns in sexual contexts [14,15,16].

In addition to conceptual issues, analyzing this phenomenon in Brazil requires consideration of structural and institutional factors. The country has a universal public health system, the Brazilian Unified Health System (SUS), grounded in the principles of universality, equity, and comprehensiveness [17]. However, the implementation of these principles faces important challenges, particularly regarding access for vulnerable populations, such as MSM and individuals who use substances in sexual contexts [13].

Available evidence [18] indicates that individuals who engage in chemsex or SDU in Brazil face multiple barriers to accessing healthcare services, including stigma and institutional discrimination, limited training of healthcare professionals, the absence of structured harm reduction approaches, and limited availability of culturally competent services [17,18]. These barriers are further exacerbated by persistent social inequalities, regional disparities, and shifts in public policies, which directly affect the organization and delivery of care.

Additionally, national scientific production on this topic remains incipient and is predominantly focused on biomedical outcomes [6], such as HIV and other sexually transmitted infections. Although these aspects are central, there is an important gap in the investigation of the psychosocial, cultural, and structural dimensions of the phenomenon, including healthcare access trajectories, users’ experiences, and the organization of care networks.

An expanded conceptual approach is therefore essential, recognizing SDU as the overarching concept encompassing diverse populations, substances, motivations, and sexual contexts, while understanding chemsex as one specific manifestation of this broader phenomenon. This perspective enables a more accurate and context-sensitive understanding of sexualized substance use in Brazil, supporting the development of public policies, healthcare practices, and harm reduction strategies that are more inclusive, effective, and equitable.

Thus, this scoping review aims to map and synthesize the available evidence on access to healthcare services among individuals who engage in SDU/chemsex in Brazil, with an emphasis on identifying barriers, gaps, and structural inequities. By incorporating an expanded perspective that integrates the concepts of chemsex and SDU, this study seeks to contribute to the development of more inclusive, context-sensitive, and equity-oriented health responses.

## 2. Materials and Methods

This scoping review was conducted based on the methodology proposed by Arksey and O’Malley [18], expanded by the recommendations of the Joanna Briggs Institute [19,20]. The protocol of this study was previously published in a scientific journal in 2025 and underwent peer review (https://doi.org/10.3390/nursrep15100353).

The study design, a non-clinical research approach as described by Brun and Zuge [21], was structured using the mnemonic framework Population, Concept, and Context (PCC) to guide data collection and support the identification of key topics, in accordance with the Joanna Briggs Institute (JBI) Manual [20]. This strategy was adopted to guide the research question of the scoping review [22].

In this study, the population includes all individuals engaged in chemsex or any form of sexualized drug use; the Concept is access to healthcare for individuals who engage in chemsex in Brazil; and the Context is Brazil. By aligning the key PCC elements with the defined objectives, the main research question of this scoping review was formulated as follows: What is known about access to healthcare among individuals who engage in chemsex in Brazil? The specific questions were: What are the healthcare needs of individuals who engage in chemsex and healthcare professionals regarding access to health services in Brazil? What existing barriers and facilitators affect access to healthcare services for individuals who engage in chemsex in Brazil? What specific services and/or resources are available within the Brazilian health system to provide care for individuals who engage in chemsex?

### 2.1. Eligibility Criteria

The eligibility criteria for the articles were as follows: studies published between 2014 and 2025; written in English, Portuguese, or Spanish; observational or experimental studies with qualitative, quantitative, or mixed-method designs; available in full text; conducted in Brazil; including individuals who engage in chemsex or sexualized drug use; and articles describing the use of psychoactive substances in sexualized contexts and/or the practice of chemsex, or providing recommendations to improve approaches to chemsex within Brazilian public health, as well as addressing barriers and gaps in access to the public healthcare system for individuals who engage in chemsex. Published literature was considered. The exclusion criteria were as follows: incomplete articles, studies in the project phase or without results, studies whose focus did not correspond to the research question, grey literature, editorials, and opinion articles.

Although community reports, dissertations, technical documents, and other forms of grey literature may include relevant initiatives, especially in the context of individuals who engage in chemsex, this review was restricted, as previously defined in the protocol, to the inclusion of peer-reviewed primary studies indexed in scientific databases. This methodological decision was made to ensure greater standardization in the quality and reporting of methods, as well as to guarantee transparency, traceability, and reproducibility in the study search and selection process.

Considering the exploratory nature of scoping reviews, it is acknowledged that the exclusion of grey literature may have limited the scope of the findings, particularly with regard to community experiences, institutional practices, and interventions not yet systematized in the scientific literature. However, this delimitation was adopted to reduce methodological heterogeneity, minimize biases resulting from the absence of peer review, and enable greater comparability among the included studies, while maintaining consistency with the criteria previously established in the review protocol.

### 2.2. Search Strategy

The search for scientific literature was conducted in journals indexed in the databases MEDLINE/PubMed, Embase, Scopus, SciELO, LILACS, and PsycINFO. The initial search strategy was developed in PubMed using the PCC framework and was based on relevant keywords combined with the Boolean operators “AND” and “OR.” This initial strategy was subsequently adapted for each selected database, taking into account the specific indexing terms, search language, and system characteristics of each platform. The final strategies were reviewed and refined with the support of a specialized librarian to ensure both precision and comprehensiveness. The search strategies were systematically documented, including information on the date of execution, the databases consulted, the combinations of terms used, the application of filters, and the number of studies retrieved (Supplementary Material S1).

For the purposes of this review, an operational definition of chemsex was adopted as the intentional use of psychoactive substances in sexual contexts with the aim of intensifying, prolonging, or facilitating the sexual experience. Studies that explicitly addressed chemsex or sexualized drug use were included, as well as those that, even without using this terminology, clearly described the association between the use of licit and illicit substances and sexual practices in the analyzed context. On the other hand, studies that addressed substance use exclusively in a general manner, without any mention or description of its association with sexual practices, were excluded. This criterion was defined to avoid undue broadening of the concept of interest and to ensure greater consistency in the composition of the sample.

For the purposes of this review, an operational definition of chemsex was adopted as the intentional use of psychoactive substances in sexual contexts with the aim of enhancing, prolonging, or facilitating the sexual experience. Studies that explicitly addressed chemsex or sexualized drug use (SDU) were included, as well as studies that, although not using this terminology, clearly described an association between the consumption of licit or illicit substances and sexual practices. Accordingly, the included studies were analyzed according to two levels of evidence: those that directly investigated the phenomenon of chemsex/SDU and those that provided indirect contextual evidence regarding substance use in sexual settings. Conversely, studies that exclusively addressed substance use in general, without any mention or description of its association with sexual practices, were excluded. This criterion was adopted to avoid an undue broadening of the concept of interest, ensure greater consistency in the composition of the sample, and enable a more appropriate interpretation of the findings in light of the different approaches represented in the literature.

The database searches were conducted in the second half of 2025 and were properly documented with detailed information regarding the databases consulted, dates of execution, and search strategies employed, including the combination of descriptors and/or keywords, the application of limits, and the number of records identified.

### 2.3. Study Selection

**Figure 1:**
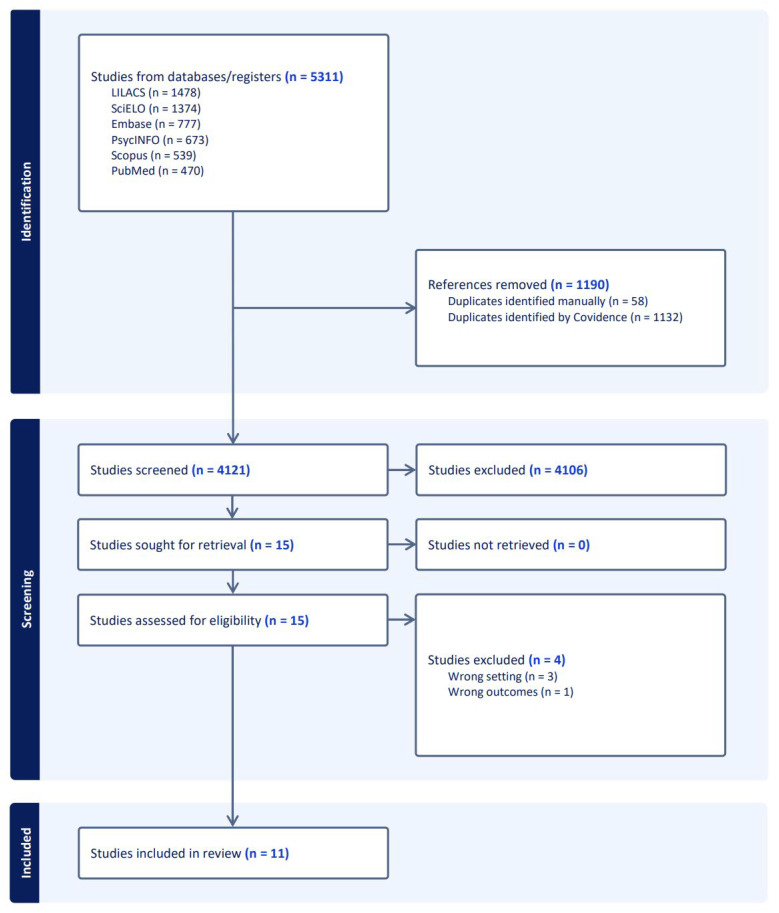
Flowchart illustrating the study selection and extraction process, developed based on the PRISMA-ScR flow [23].

The retrieved references were exported to the Covidence© software (Veritas Health Innovation, Melbourne, VIC, Australia; available at https://www.covidence.org), in which the study selection process was conducted in two stages. Initially, titles and abstracts were screened to assess eligibility. Subsequently, articles considered potentially eligible were evaluated through full-text reading. Both stages were conducted independently by two reviewers. Discrepancies were resolved by consensus through discussion between the reviewers or with the involvement of a third reviewer. The conduct and reporting of this review followed the PRISMA-ScR recommendations [23] (Supplementary Material S2). Figure 1 presents the flowchart corresponding to the study selection process.

Essential information from each included article was collected using a data extraction form specifically developed for this review, based on the model proposed by the JBI [20], with the aim of supporting descriptive and narrative/critical analyses of the selected studies. Data extraction was performed by one reviewer and subsequently checked by a second reviewer to ensure the accuracy and consistency of the data. The extraction form included the following elements: title, authorship, year of publication, methodological design, objectives, characteristics of the study population, healthcare services involved, identified access barriers, state or region where the study was conducted, and main findings.

### 2.4. Data Extraction and Analysis

After consolidating the extracted data, the results were organized into analytical categories developed to address the guiding questions of this review, based on an in-depth and iterative reading of the findings from the included studies, without prior definition based on a specific theoretical framework. This process allowed for the identification of patterns, convergences, and recurring themes in the data, guiding the development of the analytical categories. The main category of analysis focused on barriers to accessing healthcare services from the perspectives of individuals who engage in chemsex within the context of the Brazilian health system, as well as on symbolic barriers (stigma), the organization of care, gaps in professional training, and models of care (biomedical vs. integrated). No assessment of the methodological quality of the included studies was conducted, as this scoping review aimed to map the extent and characteristics of the available evidence. Although this approach is consistent with the methodology of scoping reviews, the absence of a critical appraisal of study quality limits the assessment of the robustness of the identified evidence and precludes inferences regarding the strength of the reported findings. Therefore, the results should be interpreted with caution, taking into account the potential methodological limitations of the included studies, as well as the heterogeneity of study designs, populations, and contexts investigated. Nevertheless, the mapping exercise enabled the identification of important knowledge gaps and priority areas for future research employing more rigorous methodological approaches.

### 2.5. Ethical Aspects

The present study, as a scoping review, does not require approval from a Research Ethics Committee, in accordance with the regulations established by Resolution No. 510/2016 of the National Health Council (CNS). According to this resolution, scoping reviews that involve the analysis of existing and published data do not require ethical approval, as they do not involve direct intervention with participants.

## 3. Results

A total of 5311 articles were identified in the databases. Of these, 1190 were excluded due to duplication (1132 automatically detected by Covidence© and 58 identified manually). After title and abstract screening, 4106 articles were excluded, and an additional 4 were excluded after full-text review, three for not addressing the relevant topic, that is, not addressing healthcare access or sexualized substance use, and one for not presenting results relevant to the study’s guiding questions. At the end of this process, 11 articles were selected for inclusion in the study (Figure 1).

### 3.1. Characterization of the Studies

A total of 11 studies were included in this review. Table 1 presents a synthesis of the main characteristics of the included studies, including authors, objectives, methodology, and participant profiles. Regarding methodological design, 10 studies (90.9%) adopted a quantitative approach and 1 (9.1%) a qualitative approach, with no mixed-method studies identified.

**Figure 2:**
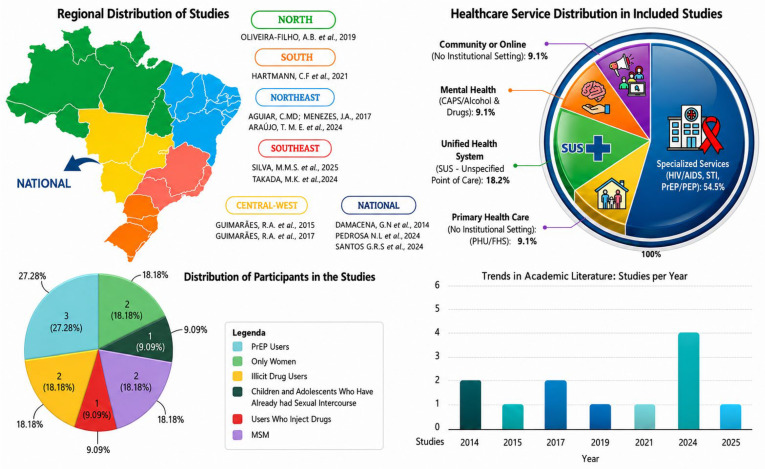
Temporal and geographic distribution and participant profile of the included studies. Notes: Damacena, G.N. et al., 2014 [24]; Guimarães, R.A. et al., 2015 [25]; Guimarães, R.A. et al., 2017 [26]; Aguiar, C.M.D; Menezes, J.A., 2017 [27]; Oliveira-Filho, A.B. et al., 2019 [28]; Hartmann, C.F. et al., 2021 [29]; Araújo, T.M.E. et al., 2024 [30]; Pedrosa, N.L. et al., 2024 [31]; Santos, G.R.S. et al., 2024 [32]; Takada, M.K.S. et al., 2024 [33]; Silva, M.M.S. et al., 2025 [34].

Studies on chemsex and healthcare access in Brazil remain scarce and unevenly distributed over time, with a greater concentration of publications in recent years. The earliest study identified was published in 2014, followed by one study in 2015. In 2017, two studies were published. After this period, scientific production remained limited, with one study published in 2019 and one in 2021. From 2024 onward, however, there was a marked increase in publications, with four studies identified in a single year, representing the period with the highest concentration of evidence included in this review. One additional study was identified in 2025, as shown in Figure 2.

Regarding geographic distribution, the studies were predominantly concentrated in large urban centers and capital cities. Two studies were conducted in the Southeast region, two in the Central-West region, and two in the Northeast region. One study was conducted in the South region (Rio Grande do Sul), while one multicenter study involved states in the North region, including Amazonas and Amapá. In addition, three studies had nationwide coverage without specific state-level delimitation.

The included studies addressed topics such as patterns of substance use, healthcare access, sexual and reproductive health, sexually transmitted infections, PrEP, risk behaviors, mental health, and health policies. Regarding study populations, two studies focused exclusively on women, including female sex workers and women who use crack. One study included people who inject drugs, two included people who use illicit drugs, one involved children and adolescents who reported previous sexual intercourse, two focused on men who have sex with men (MSM), and three included PrEP users. No studies involving healthcare professionals were identified.

To facilitate interpretation of the evidence, the studies were classified according to the nature of their contribution to the review. Four studies specifically investigated chemsex, whereas the remaining studies investigated sexualized drug use, providing contextual evidence related to substance use, sexual practices, STI vulnerability, PrEP use, and healthcare access in populations relevant to the Brazilian context.

Regarding gender identity and sexual orientation, most studies focused on cisgender gay men or MSM. Two studies included both gay and bisexual men, while only two studies more broadly included LGBTQIA+ populations, with one incorporating transgender participants as a subgroup. In one study, gender identity and sexual orientation were reported in an aggregated manner without further specification. These findings reveal important gaps in the Brazilian literature, particularly regarding transgender, non-binary, and female populations, whose experiences remain largely underrepresented in studies addressing chemsex and healthcare access.

With respect to sociodemographic characteristics, most studies reported a predominance of White participants, whereas a smaller number identified a predominance of mixed-race or Black participants. Two studies did not report race or ethnicity. Higher educational attainment was the most frequently reported educational profile, although some studies included participants with incomplete higher education or secondary education. Similarly, middle-income participants predominated across the studies, although lower- and upper-middle-income groups were also represented. Some studies did not provide sufficient information regarding educational level or income.

The limited number of studies and their concentration in large urban centers and specialized services suggest that the available evidence may not adequately represent experiences occurring in smaller municipalities, rural settings, or regions with limited healthcare infrastructure. Furthermore, because most of the included studies provided indirect contextual evidence rather than directly investigating chemsex or sexualized drug use, the findings should be interpreted with caution. Together, these factors reinforce the uneven geographic, population, and conceptual coverage of the current literature and highlight the need for future research involving more diverse populations, settings, and approaches.

### 3.2. Characterization of Chemsex in Brazil

**Figure 3:**
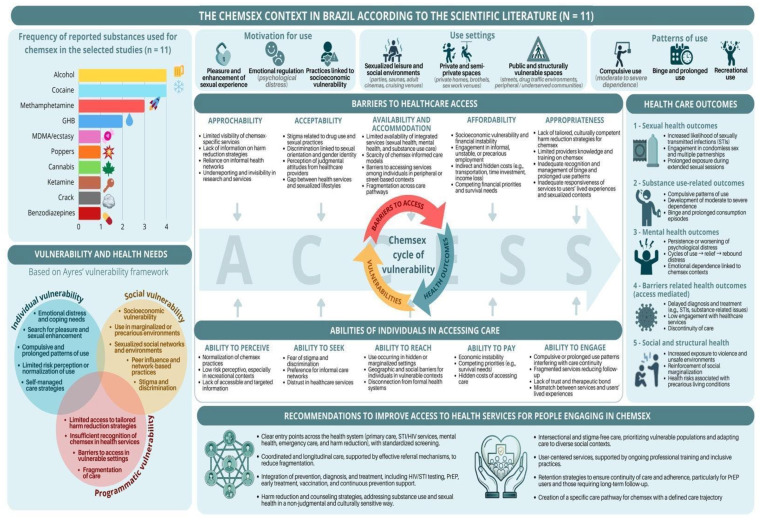
The VIP-SDU/Chemsex Model: A Multilevel Framework of Vulnerabilities, Healthcare Access, and Health Outcomes in Brazil.

Figure 3 presents an integrative synthesis of chemsex in Brazil, as evidenced in the included literature, articulating multiple dimensions: patterns and contexts of use, motivations, vulnerabilities, access barriers, individuals’ capacities, and health outcomes within a single explanatory model.

Regarding the substances used, the studies were classified according to the primary pattern of use reported. Four studies (36.4%) predominantly described the combined use of alcohol and cocaine, followed by methamphetamine in three studies (27.3%) and GHB/GBL in two studies (18.2%). In addition, one study focused on MDMA/ecstasy and poppers (9.1%), and another focused on other substances, such as cannabis, ketamine, crack, and benzodiazepines (9.1%).

In terms of motivations for use, four studies (36.4%) indicated increased sexual pleasure and disinhibition as the primary motivation, three studies (27.3%) pointed to the facilitation of social bonding, intimacy, and a sense of belonging, two studies (18.2%) associated use with coping with stigma, loneliness, or psychological distress, one study (9.1%) linked motivation to the maintenance of sexual performance, and one study (9.1%) did not specify the primary motivation. Regarding the context of use, five studies (45.5%) described private settings (homes, closed gatherings), three studies (27.3%) reported private sex parties or clubs, two studies (18.2%) indicated use mediated by dating applications, and one study (9.1%) described mixed or unspecified settings.

Regarding risk behaviors, five studies (45.5%) highlighted condomless sex and multiple partners as the primary risk behavior, three studies (27.3%) emphasized polysubstance use associated with sexual activity, two studies (18.2%) reported high exposure to STIs/HIV due to informal work, and one study (9.1%) addressed risk behaviors in an aggregated manner without specific detail.

With regard to sexually transmitted infections, the studies were classified according to the primary condition reported. It was observed that four studies (36.4%) focused primarily on HIV, while three (27.3%) addressed multiple concurrent STIs. Syphilis was the primary condition in two studies (18.2%), followed by viral hepatitis in one study (9.1%). Other STIs, such as HPV and Mpox (formerly known as monkeypox), were reported in one study (9.1%).

The organization of the phenomenon around a cycle of vulnerability is noteworthy, in which individual, social, and programmatic conditions not only precede but are also intensified by barriers to access and negative health outcomes. In this sense, chemsex is represented as a dynamic and complex phenomenon that extends beyond the behavioral dimension and is deeply influenced by social determinants, structural contexts, and the way healthcare services are organized and respond to these demands.

Additionally, the figure highlights that barriers to care and individuals’ capacities to access services are not isolated elements but interdependent components that influence continuity of care and the effectiveness of health responses. This analytical framework makes it possible to identify gaps in care and reinforces the need for integrated, user-centered approaches that are sensitive to the specific contexts in which chemsex occurs.

### 3.3. Access to Health Care (Descriptive Data)

The findings on healthcare access, when articulated with the model presented in Figure 3, allow this phenomenon to be understood as a dynamic process resulting from the interaction between the organization of services and individuals’ capacities to recognize needs, seek care, and remain engaged in care.

Regarding the healthcare services presented in Figure 2, six studies (54.5%) were conducted in specialized services focused on HIV/acquired immunodeficiency syndrome (AIDS), sexually transmitted infections (STIs), and prevention strategies such as pre-exposure prophylaxis (PrEP) and post-exposure prophylaxis (PEP). These services included outpatient clinics, Testing and Counseling Centers (CTA), and Specialized Care Services (SAE). Two studies (18.2%) were conducted within the Brazilian Unified Health System (SUS), specifically in Basic Health Units (UBS), although without specification of the level of care. One study (9.1%) was conducted in Primary Health Care (PHC), one (9.1%) in mental health services, specifically Psychosocial Care Centers (CAPS), and one (9.1%) in a community or online context without a defined institutional setting.

This distribution highlights the central role of specialized services in care provision, in contrast to the limited participation of services that could promote continuity and coordination of care.

Regarding health needs, four studies (36.4%) pointed to combination prevention and STI/HIV care, three studies (27.3%) highlighted mental health care, two studies (18.2%) emphasized harm reduction strategies, one study (9.1%) indicated the need for welcoming care and longitudinal engagement, and one study (9.1%) did not specify priorities. As represented in Figure 3, these needs are distributed across different dimensions of care and require integrated responses within the healthcare network.

Regarding the dimensions of healthcare access, four studies (36.4%) analyzed the availability and provision of services, three studies (27.3%) addressed acceptability, two studies (18.2%) discussed accessibility, one study (9.1%) evaluated effective use, and one study (9.1%) addressed access more broadly. In Figure 3, these dimensions are presented as interrelated components that influence care pathways and continuity of care.

Regarding difficulties and barriers to access, four studies (36.4%) identified stigma, discrimination, and moral judgment, three studies (27.3%) pointed to gaps in professional training and institutional unpreparedness, two studies (18.2%) highlighted fragmentation of care, one study (9.1%) indicated structural and organizational barriers, and one study (9.1%) described fear of exposure and breaches of confidentiality. As synthesized in Figure 3, these barriers operate interdependently and affect different stages of access, influencing engagement, retention, and the effectiveness of the care provided.

### 3.4. Analytical Categories

Based on the synthesis of findings presented above and considering the overall landscape of the analyzed studies, analytical categories were developed to address the main and specific research questions of this review, related to health needs, barriers and facilitators of access, and the services and resources available within the healthcare system. The identified categories were: structural barriers to access; symbolic barriers (stigma); organization of care; gaps in professional training; models of care (biomedical vs. integrated).

Structural barriers to access were identified in some of the studies, particularly in relation to accessibility and the organization of services. Two studies (18.2%) directly analyzed geographic, economic, and structural barriers affecting access to healthcare. In addition, one study (9.1%) highlighted difficulties such as restricted access, incompatible service hours, difficulty scheduling appointments, and discontinuity of care. It was also observed that most studies were conducted in specialized HIV/AIDS, STI, and PrEP/PEP services (54.5%), with a lower representation of studies in PHC (9.1%) and in mental health services (9.1%), indicating a limited distribution of points of care.

Symbolic barriers were the most frequently reported, identified in four studies (36.4%) as stigma, discrimination, and moral judgment related to sexual orientation, gender identity, and substance use. In addition, one study (9.1%) identified fear of exposure, breaches of privacy, or loss of confidentiality as limiting factors for access to healthcare services. In two studies (18.2%), substance use was associated with coping with stigma, loneliness, or psychological distress.

The organization of care was characterized by fragmentation of services, identified in two studies (18.2%), and marked by a lack of integration among sexual health, mental health, and substance use care. The distribution of studies also showed a concentration in specialized HIV/AIDS, STI, and PrEP/PEP services (54.5%), with lower representation in other levels of care, such as PHC (9.1%) and mental health services (9.1%). Regarding health needs, one study (9.1%) highlighted the importance of welcoming care and longitudinal engagement, while others identified demands related to combination prevention, mental health, and harm reduction.

Gaps in professional training were identified in three studies (27.3%), which pointed to a lack of training among healthcare professionals and institutional unpreparedness to address the specificities of chemsex. These studies highlighted difficulties related to managing issues involving substance use, sexual practices, and diversity in sexual orientation and gender identity. In addition, no studies were identified that included healthcare professionals as participants.

The results indicate a predominance of studies conducted in specialized HIV/AIDS, STI, and PrEP/PEP services (54.5%), focusing on topics such as sexually transmitted infections, combination prevention, and risk behavior. Regarding health needs, four studies (36.4%) identified demands related to STI/HIV prevention and care, while three (27.3%) highlighted mental health, and two (18.2%) addressed the need for harm reduction strategies. These findings highlight the coexistence of different dimensions of care, including biomedical aspects as well as other needs related to mental health and substance use.

Collectively, these findings reveal that healthcare access among individuals who engage in chemsex is shaped by the interaction of individual, social, and programmatic factors. To better understand these relationships, we developed the Vulnerabilities, Inequities, and Programmatic Responses in Chemsex (VIP-SDU/Chemsex) Model, which is discussed below in light of established theoretical frameworks.

## 4. Discussion

The analysis of the literature indicates that chemsex is an emerging phenomenon in Brazil, with greater expression among MSM and cisgender men, but still marked by limited visibility in healthcare systems, scientific production, and public policies [26]. This gap extends beyond the absence of data and reflects how chemsex is insufficiently recognized within care pathways. As illustrated in Figure 3, access to care is shaped by the interaction between institutional barriers, individual capacities, and health outcomes, reinforcing the need for integrated approaches.

The findings of this review support the development of the VIP-SDU/Chemsex Model, which integrates vulnerability theory, syndemic theory, intersectionality, and healthcare access frameworks to explain how healthcare access is produced within the context of SDU/chemsex in Brazil. Rather than identifying isolated barriers, the review demonstrates that individual practices, social inequalities, and programmatic limitations interact dynamically, producing cumulative vulnerabilities and shaping health outcomes. The model therefore provides an analytical framework for understanding SDU/chemsex not merely as an individual behavior, but as a phenomenon embedded within broader social, institutional, and structural contexts.

### 4.1. Theoretical Foundations of the VIP-SDU/Chemsex Model

The Vulnerabilities, Inequities, and Programmatic Responses in SDU/Chemsex (VIP- SDU/Chemsex) Model is proposed as a preliminary conceptual framework developed by the authors to organize and interpret the findings of this scoping review. Rather than representing an empirically validated model or establishing causal relationships, the framework integrates the evidence identified in this review with established theoretical perspectives and findings from our broader research program on SDU/chemsex, healthcare access, HIV prevention, health literacy, and healthcare pathways among key populations in Brazil [14,15]. It is intended to support future research, guide hypothesis generation, and inform the development of comprehensive healthcare strategies.

The first theoretical foundation of the framework is the Vulnerability Framework proposed by Ayres et al. [35], which conceptualizes vulnerability as a multidimensional phenomenon encompassing individual, social, and programmatic dimensions. The studies included in this review identified elements consistent with these dimensions, including substance use patterns and sexual practices (individual vulnerability), stigma and discrimination (social vulnerability), and fragmentation of healthcare services together with insufficient professional preparedness (programmatic vulnerability). These findings were used to organize, rather than validate, the proposed conceptual structure.

The framework is also informed by Syndemic Theory, which proposes that health conditions may interact under conditions of social inequality. Across the included studies, recurring themes included sexually transmitted infections, mental health challenges, substance use, stigma, and barriers to healthcare access. Although the available evidence does not permit causal inferences regarding these interactions, the syndemic perspective offers a useful theoretical lens for interpreting their co-occurrence.

Additionally, the VIP- SDU/Chemsex Model incorporates principles from Intersectionality Theory. The review identified disparities related to sexual orientation, gender identity, race, socioeconomic position, and social exclusion. These factors appear to intersect in shaping experiences related to healthcare access and vulnerability. Furthermore, the limited representation of women, transgender, and non-binary individuals in the available literature highlights important knowledge gaps and reinforces the need for intersectional approaches in future research.

Finally, the framework draws upon Levesque’s Framework [36] for Access to Healthcare, which conceptualizes access as a dynamic interaction between health system characteristics and individuals’ abilities to perceive health needs, seek care, reach services, pay for care, and engage with healthcare providers. The evidence synthesized in this review suggests that barriers such as stigma, fear of disclosure, fragmented services, limited professional preparedness, and insufficient integration of care may influence several dimensions of healthcare access described by this framework.

Taken together, these theoretical perspectives, alongside the evidence synthesized in this review and complementary findings from our ongoing research program, informed the development of the VIP- SDU/Chemsex Model. The framework should therefore be interpreted as a conceptual representation of factors that may influence healthcare access among individuals who engage in SDU/chemsex in Brazil, rather than as a validated explanatory model. Future empirical studies are needed to examine, refine, and validate its components and relationships across different healthcare settings and populations.

### 4.2. Sociodemographic Characteristics

From a sociodemographic perspective, although sexualized drug use is internationally recognized as a relevant public health issue, in Brazil, there is still a significant lack of systematic investigations and specific guidelines [1]. This gap is directly related to the dimension of approachability, as the limited visibility of chemsex in health services and the absence of structured harm reduction strategies contribute to its under-recognition. As a result, care tends to be poorly adapted to the needs of this population, negatively affecting access, prevention, and continuity of care [27].

The concentration of studies in large urban centers, particularly in the Southeast, Northeast, and Central-West regions, reflects both disparities in research production and inequalities in service provision. Regions with less developed healthcare infrastructure tend to have fewer specialized services, which limits access to and continuity of care [28]. Additionally, the absence of studies in smaller municipalities restricts the understanding of chemsex in diverse territorial contexts and reinforces the invisibility of experiences outside major urban centers [29].

Regarding population profiles, the predominance of cisgender MSM, combined with the limited inclusion of women, transgender, and non-binary individuals, reveals an important gap in the literature. This absence reflects not only methodological limitations but also broader social processes that influence which experiences are documented and prioritized in research. The invisibility of women, for instance, does not indicate the absence of chemsex practices, but rather the historical marginalization of their sexual experiences [37]. As discussed in gender studies, these dynamics are shaped by power relations that regulate which bodies and practices are considered legitimate objects of scientific inquiry [38,39].

Women may experience substance use in contexts marked by gender inequalities, economic dependence, and exposure to violence. These conditions are associated with higher levels of psychological distress and may contribute to substance use as a coping strategy [38]. Evidence shows that experiences of physical, psychological, or sexual violence are associated with a higher prevalence of problematic substance use among women, increasing health risks [37]. These findings highlight the need for intersectoral approaches that integrate health, social protection, and gender-based violence policies [39].

Similarly, transgender individuals remain largely absent from the analyzed studies. This underrepresentation reflects structural barriers to accessing both healthcare and research participation, including discrimination, economic marginalization, and limited access to education and digital resources [40]. These factors hinder engagement and contribute to the scarcity of data, limiting the understanding of their specific health needs and reinforcing the importance of more inclusive research strategies [41]. This invisibility suggests that the mechanisms that produce social exclusion also influence the production of scientific knowledge, resulting in a dual vulnerability: reduced access to services and lower representation in the evidence base that informs policies and healthcare practices.

### 4.3. Chemsex

The findings indicate that, although substances commonly associated with chemsex in the international literature, such as methamphetamine, GHB/GBL, MDMA, and poppers, are present in Brazil, there are important contextual differences. In particular, the combined use of alcohol and cocaine appears as a central pattern in the Brazilian context [39]. This reflects factors such as availability, cultural integration, and cost, as Brazil occupies a strategic position in drug trafficking routes.

In contrast to European and North American contexts, where methamphetamine and GHB/GBL are more central, the Brazilian scenario suggests a broader understanding of chemsex that includes substances traditionally associated with nightlife and recreational use [41]. This reinforces the importance of analyzing chemsex as a context-dependent phenomenon, shaped by social, economic, and cultural factors.

Motivations for chemsex are multifaceted. The literature consistently highlights increased sexual pleasure, disinhibition, and prolonged sexual experiences as key drivers, alongside the pursuit of intimacy, belonging, and coping with psychological distress [40]. In this sense, chemsex may function both as a facilitator of social interaction and as a strategy for managing emotional and relational challenges.

The contexts of use also contribute to this dynamic. Most studies describe private environments, such as homes or organized parties, often mediated by digital platforms, where practices tend to be prolonged and less regulated [18]. The frequent use of multiple substances reflects the functional nature of chemsex, with different drugs combined to sustain extended sexual encounters, thereby increasing exposure to health risks [41].

### 4.4. Access to Healthcare

#### 4.4.1. Symbolic Barriers

Barriers to healthcare access are strongly influenced by moral judgments and stigmatizing attitudes embedded in healthcare practices [42]. These dynamics shape how sexualized drug use is perceived and addressed within services, often leading to reduced openness in communication and limiting the recognition of users’ needs [4]. As a result, care may become less responsive and less tailored to the complexity of these experiences [43].

These barriers directly affect acceptability, influencing trust, engagement, and continuity of care [17]. In parallel, structural issues, such as limited service integration, affect availability and appropriateness, making it more difficult to address overlapping needs related to sexual health, substance use, and mental health [44]. Economic instability further impacts affordability, restricting sustained access to services [45].

Access is also shaped by individuals’ capacities to perceive, seek, reach, pay for, and engage with care [45]. The predominance of specialized service use, rather than primary healthcare, reflects limitations in these capacities, particularly regarding trust and the perceived quality of care [34].

Racial inequalities add another layer of complexity. The lower representation of Black individuals in the analyzed studies suggests barriers to both healthcare access and research participation, influenced by structural racism [46]. These findings highlight the importance of considering the intersection of race, sexual orientation, gender identity, and substance use in understanding healthcare inequalities [3].

#### 4.4.2. Structural Barriers

The predominance of studies focused on STIs, PrEP, and risk behaviors suggests that Brazilian scientific production remains strongly influenced by a biomedical framework, while aspects related to the organization of care, users’ experiences, and professional practices remain relatively underexplored. The thematic diversity of the studies, including substance use, sexual health, mental health, and health policies, highlights the multifaceted nature of chemsex [47]. However, few studies address the organization of care or include healthcare professionals, limiting the understanding of institutional barriers.

Professional preparedness emerges as a key issue. Limited training on sexualized drug use and related topics affects the quality of care and the ability to respond to complex needs [43]. These barriers are not limited to service availability but are also related to how care is structured and delivered [38].

The concept of a “cycle of vulnerability,” illustrated in Figure 3, helps explain how social conditions, institutional barriers, and health outcomes reinforce each other. Inadequate responses can perpetuate exposure to risks while limiting access to effective care strategies [45]. The high prevalence of sexually transmitted infections in contexts of prolonged sexual activity and substance use further highlights the interconnection between pleasure, risk, and care [48].

These findings point to the need to strengthen public policies and improve care models, with an emphasis on integration, professional training, and more coordinated responses.

#### 4.4.3. Organization of Care

Although harm reduction is a well-established approach in substance use, its application in the context of SDU/chemsex remains limited. It is often restricted to drug use, without fully addressing the sexual dimension of these practices [29]. This separation reduces the effectiveness of care and limits its ability to respond to real needs.

Integrating harm reduction with sexual health is essential. Such integration allows for more realistic and person-centered approaches, focusing on prevention, autonomy, and health promotion [41]. This is particularly relevant in chemsex, where substance use and sexual practices are closely interconnected.

Primary healthcare plays a strategic role in this context. Its emphasis on longitudinal care and accessibility supports the development of therapeutic relationships and continuous monitoring [49]. For individuals who engage in SDU/chemsex, this continuity is essential due to the complexity of their health needs [11].

Nursing professionals are central to this process. Their role in care coordination, education, and patient support enables more comprehensive interventions. Harm reduction, as a guiding principle, supports strategies focused on minimizing risks without requiring abstinence [50]. These include sexual health counseling, STIs prevention, safer substance use guidance, and early identification of complications [51].

#### 4.4.4. Gaps in Professional Training

The preference for specialized services reflects perceived limitations in primary healthcare. These services are often seen as better prepared to address issues related to sexual diversity and substance use [11]. In contrast, primary care is frequently perceived as less equipped, particularly due to gaps in training and concerns about confidentiality and judgment.

These limitations affect the use of primary healthcare services and contribute to the concentration of care in specialized centers, often located in large cities [52]. This centralization can limit access to and continuity of care, especially for individuals in underserved regions.

Strengthening professional training is therefore essential. This includes not only technical knowledge but also communication skills and attitudes that support inclusive and respectful care. Nursing plays a key role in mediating interprofessional care and improving service integration, contributing to more effective and equitable care pathways [52,53]. The role of nursing in coordinating care across the healthcare network is particularly noteworthy, as it facilitates integration between Primary Health Care services, specialized STI/HIV services, mental health services, and services dedicated to the care of people who use alcohol and other drugs. Through the coordination of interprofessional care, longitudinal follow-up, and timely referrals across different levels of care, nurses can help minimize the fragmentation of care identified in the studies, promoting greater continuity, comprehensiveness, and effectiveness of health interventions directed toward individuals who engage in SDU/chemsex [49].

#### 4.4.5. Models of Care

The health needs of individuals who engage in SDU/chemsex are predominantly addressed through biomedical approaches, particularly focused on STI/HIV prevention and treatment [53]. While important, this focus does not fully address broader needs related to mental health, social support, and harm reduction.

According to the vulnerability framework, these needs involve individual, programmatic, and social dimensions [54]. Addressing them requires more than the availability of services; it depends on the system’s ability to provide integrated and responsive care [44].

Patterns of sexual behavior and substance use described in the studies highlight the complexity of chemsex practices. The combination of multiple partners, condomless sex, and substance use creates conditions that facilitate STIs transmission [48]. These practices are embedded in specific social networks, reinforcing their continuity [43].

The high prevalence of infections such as HIV, syphilis, and viral hepatitis underscores the public health relevance of SDU/chemsex [55]. Delays in diagnosis and fragmented care can contribute to ongoing transmission [46]. These findings highlight the limitations of individual-focused interventions and the need for integrated and structural responses [52].

Socioeconomic factors, such as informal work, further influence health risks and access to care. Economic instability can limit the ability to negotiate safer practices and sustain engagement with services [43]. These dynamics demonstrate that health behaviors are shaped by broader social conditions, not only individual choices [10].

Finally, the limited focus on harm reduction strategies in the literature represents an important gap. While mental health outcomes are frequently discussed, fewer studies address practical interventions that could reduce risks. Expanding this perspective is essential for developing more effective and comprehensive care models [30,40].

#### 4.4.6. The VIP- SDU/Chemsex Model: A Multilevel Framework of Vulnerabilities and Healthcare Access

Based on the integration of the evidence synthesized in this scoping review, established theoretical perspectives, and complementary findings from our broader research program on SDU/chemsex and healthcare in Brazil, we propose the VIP-SDU/Chemsex Model (Vulnerabilities, Inequities, and Programmatic Responses in SDU/Chemsex) as a preliminary conceptual framework. The model is intended to organize the available evidence and support the interpretation of healthcare access among individuals who engage in SDU/chemsex rather than to represent an empirically validated or causal model.

The VIP- SDU/Chemsex Model is organized as a multilevel conceptual framework comprising three interconnected domains: (1) structural and symbolic vulnerabilities, (2) programmatic responses within the healthcare system, and (3) individual capacities related to healthcare access. These domains are proposed as interacting dimensions that may influence experiences of healthcare access and health outcomes among individuals who engage in SDU/chemsex.

The framework includes five conceptual components: (i) structural vulnerabilities, including socioeconomic inequalities, regional disparities, and service availability; (ii) symbolic vulnerabilities, such as stigma, discrimination, and moral judgment; (iii) programmatic vulnerabilities, including fragmentation of care, insufficient professional preparedness, and the limited availability of integrated services; (iv) healthcare access capacities, encompassing individuals’ abilities to perceive health needs, seek care, reach services, engage with healthcare, and maintain continuity of care; and (v) health-related outcomes potentially associated with SDU/chemsex practices. These components were derived from recurring themes identified in the included studies and interpreted in light of the theoretical frameworks adopted.

Rather than implying causal pathways, the proposed framework suggests that structural, symbolic, and programmatic vulnerabilities may interact in ways that influence healthcare access. Likewise, integrated, stigma-informed, and person-centred healthcare responses may contribute to improving access and continuity of care. These propositions should be interpreted as conceptual hypotheses requiring future empirical investigation.

The framework adopts a multilevel perspective encompassing macro-level determinants (social and structural conditions), meso-level factors (health system organization), and micro-level experiences (individual behaviors and healthcare interactions). This perspective supports a broader understanding of SDU/chemsex by considering institutional, organizational, and social contexts in addition to individual behaviors.

From a health systems perspective, the framework highlights potential areas for strengthening the organization of care within the Brazilian Unified Health System (SUS), including greater integration between primary healthcare, specialized sexual health services, mental healthcare, substance use services, and harm reduction initiatives. These implications should be understood as conceptual directions derived from the available evidence rather than as evidence-based recommendations.

Similarly, from a nursing perspective, the VIP- SDU/Chemsex Model may serve as a conceptual tool to support comprehensive assessment, care planning, harm reduction practices, and care coordination for individuals who engage in SDU/chemsex. The framework may also assist in identifying multilevel vulnerabilities and informing educational initiatives for healthcare professionals, although its practical applicability requires future evaluation.

Although the proposed VIP-SDU/Chemsex Model has not been empirically validated, it should be interpreted as a preliminary conceptual framework developed through the integration of the evidence synthesized in this scoping review, established theoretical perspectives, and complementary findings from our broader research program on SDU/chemsex, healthcare access, and HIV prevention in Brazil [14,15]. As such, the model is intended to organize current knowledge, generate hypotheses, and support future research rather than establish causal relationships or validated pathways.

Within this perspective, the findings synthesized in this review suggest that strengthening integrated responses to SDU/chemsex within the Brazilian Unified Health System (SUS) may represent an important direction for future policy development, service organization, and research. In particular, the development and evaluation of care pathways integrating primary healthcare, specialized sexual health services, mental healthcare, substance use services, and harm reduction strategies deserve further investigation as potential approaches to addressing the complex healthcare needs of individuals who engage in SDU/chemsex.

By integrating the Vulnerability Framework, Syndemic Theory, Intersectionality Theory, and Levesque’s Framework for Access to Healthcare, the VIP-SDU/Chemsex Model offers a conceptual lens through which the interplay between individual, social, and programmatic vulnerabilities and healthcare access can be explored. Rather than representing an empirically validated explanatory model, it provides a structured framework for organizing the currently available evidence and identifying priorities for future investigation.

Similarly, the conceptual framework may support the future development of care pathways, harm reduction strategies, continuing professional education, and public health policies aimed at improving healthcare access in SDU/chemsex contexts. However, these implications should be regarded as preliminary, given the limited number of included studies, their methodological and population heterogeneity, the predominance of indirect evidence, and the concentration of research in specific urban settings and population groups. Future empirical studies are needed to refine, validate, and assess the applicability of the proposed framework across different Brazilian contexts.

##### Limitations

This review has some limitations. The decision to include exclusively peer-reviewed primary studies may have resulted in the exclusion of grey literature, as well as institutional and community reports that often document innovative community-led initiatives, especially in the context of individuals who engage in chemsex. This limitation is particularly relevant in the context of chemsex, as community-based harm reduction initiatives, local care strategies, experiences of non-governmental organizations, and technical reports often constitute important sources of knowledge and innovation in health care, yet are not always disseminated through indexed scientific publications. In this sense, future research may benefit from incorporating a broader range of sources to capture perspectives grounded in community experience. In addition, the findings of this review are conditioned by the information reported in the included studies, which may have limited the identification of other relevant experiences while also highlighting the need to expand participatory research conducted in partnership with these populations.

It was also observed that most studies focused on individuals who engage in chemsex, providing important contributions to understanding the dynamics of this phenomenon. However, no studies were identified that explored the perspectives of healthcare professionals on chemsex or their care practices directed at this population. Similarly, there was a scarcity of investigations focusing primarily on women, suggesting a context of underrepresentation. These asymmetries indicate structural gaps in research agendas and reinforce the importance of adopting intersectional approaches.

On the other hand, these gaps themselves constitute relevant findings, as they highlight the need to diversify both care models and research approaches. In summary, although the methodological choices impose certain limitations, they also contribute to strengthening the rigor of the review and to identifying silences and inequalities in scientific production. These elements not only consolidate the current state of knowledge but also guide the development of future research that is more inclusive, intersectional, and context-sensitive.

Additionally, it is important to note that the VIP-SDU/Chemsex Model proposed in this study has not been empirically validated and should be interpreted as a theoretical-analytical synthesis constructed from the integration of the findings of this review. In this regard, its applicability and robustness should be explored in future research, particularly in empirical studies that include diverse populations and care settings.

## 5. Conclusions

Although Brazil has relevant normative frameworks, such as the National STI/HIV/AIDS Policy, the National Comprehensive Health Policy for LGBT+ People, the National Mental Health Policy, and harm reduction guidelines, the results of this review indicate that these policies are implemented in a fragmented manner and do not specifically address the particularities of SDU/chemsex. The absence of clinical care protocols, integrated care pathways, and, above all, a structured care line for individuals who engage in SDU/chemsex contributes to reliance on isolated initiatives, often restricted to specialized services or community-based projects, with limited reach and low sustainability.

The importance of continuing health education and the incorporation of this topic into health professional training curricula is evident in order to qualify care practices that are sensitive to the complexities surrounding SDU/chemsex. The still-limited incorporation of harm reduction policies in healthcare services, as well as in scientific production and research agendas, constrains the development of comprehensive and context-sensitive responses to the needs of this population.

In this context, the findings of this review, articulated with the VIP- SDU/Chemsex Model (Intersectional and Programmatic Vulnerabilities in SDU/Chemsex Care), suggest that the absence of a structured care line is not merely an organizational gap but rather an expression of programmatic vulnerabilities that restrict the recognition, integration, and effectiveness of responses within the Brazilian Unified Health System (SUS).

In this sense, the findings of the analyzed literature can support the strengthening of public policies and the reorientation of the organization of care within the SUS. This includes the development of a specific care pathway for individuals who engage in SDU/chemsex, integrated into the Health Care Network, linking primary care, specialized services, mental health, and harm reduction strategies. Such an approach supports the promotion of comprehensive, equitable, and effective care.

By integrating empirical evidence, theoretical contributions, and implications for practice, this study reaffirms SDU/chemsex as an emerging public health issue in Brazil and highlights that expanding access to care is not limited to the provision of biomedical technologies but requires institutional, policy, and educational transformations capable of addressing invisibility, stigma, and fragmentation of care, in alignment with the principles of the SUS and the promotion of health equity.

## Figures and Tables

**Table 1:** Characteristics of the studies included in the scoping review sample, in terms of authors, year of publication, geographic location of the study, objective, method, design, and participant profile.

Authors/Year/Setting	Objective	Method/Design	Participants	Evidence Type	Definition	Substances, Motivations, and Contexts Were Reported	Dimensions of Access and Barriers and Facilitators	Contribution to the VIP-SDU/Chemsex Model
DAMACENA, G.N. et al., 2014 [24], Brazil	To investigate differences in HIV-related risk practices among female sex workers according to their workplace setting, and to assess the effects of homophily on HIV prevalence estimation.	Quantitative—Cross-sectional study	Female sex workers aged ≥18 years who have exchanged sex for money in the past 4 months.	Indirect contextual evidence	Sexualized drug use	Alcohol; crack/merla; cocaine. Street and houses of prostitution. Sex work for income and drug acquisition	Accessibility; Lack of a fixed geographic linkage to healthcare services. Difficulty accessing services that operate only during regular business hours. Lack of perceived coping strategies.	Highlights structural barriers to healthcare access among socially vulnerable populations.
GUIMARÃES, R.A. et al., 2015 [25], Goiânia—GO	To investigate the prevalence and risk behaviors through self-reported sexually transmitted infections among crack users.	Quantitative- Cross-sectional study	Individuals aged 18 years or older who had used crack for at least one month prior to admission.	Indirect contextual evidence	Sexualized drug use	Crack cocaine; alcohol; cannabis; snorted cocaine. Intentional engagement in sexual activity to obtain drugs and/or financial resources for drug acquisition. Use settings not described.	Accessibility; Access barriers: Low income; incarceration history; sex work; illicit activities; social vulnerability. Lack of perceived coping strategies.	Demonstrates the interaction between substance use, sexual vulnerability, and social exclusion.
GUIMARÃES, R.A. et al., 2017 [26], Goiás—GO	To estimate the prevalence and identify factors associated with lifetime HIV testing among non-injecting drug users (NIDU).	Quantitative—Cross-sectional study	Non-injecting drug users.	Indirect contextual evidence	Sexualized drug use	Crack, cocaine; cannabis; inhalants; LSD; ecstasy (MDMA); non-injectable heroin. Recreational and compulsive use; facilitation of sexual activity; pleasure; social context. Use settings not reported.	Availability; accessibility; acceptability. Stigma; marginalization; limited availability of rapid testing; concentration of testing services in specialized facilities; low risk perception Coping strategies: HIV counseling; engagement with treatment services	Identifies programmatic barriers to HIV testing and the impact of stigma on healthcare utilization.
AGUIAR, C.M.D; MENEZES, J.A., 2017 [27], Recife—PE	To analyze the sexual experiences of young female crack users, as well as the repercussions of such practices on their lives.	Qualitative—Discourse analysis	Young female crack users.	Indirect contextual evidence	Sexualized drug use	Crack cocaine; alcohol; tobacco; cannabis; inhalants (lança-perfume/glue); cocaine. Relief of emotional distress; pleasure; escape from reality; economic survival. Streets; brothels; community settings; drug trafficking environments	Accessibility; availability. Access barriers: Stigma; discrimination; incompatible service hours; territorial barriers; social exclusion. Coping strategies: Institutional support; therapeutic relationships with healthcare professionals; motherhood; religious involvement; temporary affective/social support networks.	Provides evidence on stigma, discrimination, and the importance of supportive healthcare relationships.
OLIVEIRA-FILHO, A.B. et al., 2019 [28], North	To determine the prevalence and factors associated with HCV infection and spontaneous HCV clearance among individuals who have used illicit drugs in the Amazon region.	Quantitative—Cross-sectional study	People who use illicit drugs (PWUIDs).	Indirect contextual evidence	Sexualized drug use	Crack cocaine/oxi; cocaine; cannabis. Recreational use. Communities and environments characterized by intensive drug use.	Availability Social vulnerability; socioeconomic marginalization; limited healthcare infrastructure in the region.	Reinforces the role of socioeconomic vulnerability and regional inequities in healthcare access.
HARTMANN, C.F. et al., 2021 [29], South	To estimate the prevalence and identify factors associated with risky sexual behavior among street-connected children, adolescents, and youth.	Quantitative—Cross-sectional study	Children, adolescents, and youth (10–21 years) who reported having had sexual intercourse.	Indirect contextual evidence	Sexualized drug use	Illicit drugs. Coping with life on the streets; altered perception of reality; pleasure. Use settings: Street	Accessibility; availability. Shame in seeking healthcare services; social vulnerability.	Highlights how social vulnerability and marginalization limit access to healthcare.
ARAÚJO, T.M.E. et al., 2024 [30], Teresina—PI	To investigate sexual practices and perceptions of HIV risk among men who have sex with men (MSM), identifying associated risk factors and determinants.	Quantitative—Cross-sectional study	Men who have sex with men (MSM).	Direct contextual evidence	Chemsex: “the use of illicit drugs before or during sexual activity.”	Alcohol; cannabis. Use during sexual activity. Saunas; adult cinemas; cruising locations.	Acceptability; accessibility. Low income; poor risk perception; limited access to information, prevention resources, and healthcare services.	Contributes evidence on chemsex contexts, HIV risk perception, and barriers to prevention and care.
PEDROSA, N.L. et al., 2024 [31], Brazil	To investigate the incidence and associated factors of acquired syphilis among PrEP users in Brazil.	Quantitative—Retrospective cohort study	PrEP users.	Direct contextual evidence	Chemsex: “the use of illicit drugs before or during sexual activity.”	Poppers; cocaine; cannabis; erectile stimulants; club drugs (MDMA, GHB, ketamine, LSD, methamphetamine); alcohol. Use for sexual practices. Home settings; sexual encounters.	Availability. Lack of testing or unavailable testing; loss to follow-up.	Demonstrates the association between chemsex, STI outcomes, and gaps in continuity of care.
SANTOS, G.R.S. et al., 2024 [32], Brazil	To investigate factors associated with chemsex practices among men who have sex with men (MSM) in Brazil during the mpox outbreak.	Quantitative—Cross-sectional study	Men who have sex with men (≥18 years), residing in Brazil.	Direct contextual evidence	Chemsex: “Intentional use of drugs immediately before and/or during sexual intercourse.”	GHB/GBL; methamphetamine; mephedrone; MDMA/ecstasy; opioids; amphetamines; poppers. Enhancement of sexual pleasure; disinhibition; coping with stigma and health crises. Collective environments (saunas, parties, cinemas, sex clubs); encounters via dating apps.	Acceptability; accessibility; appropriateness. Fear of discrimination (health services, friends, and family); stigma associated with mpox.	Shows the interplay between stigma, chemsex practices, and healthcare access during public health emergencies.
TAKADA, M.K.S. et al., 2024 [33], São Paulo—SP	To assess the impact of PrEP use on users’ health-related quality of life.	Quantitative—Prospective observational cohort study	HIV-negative participants aged ≥18 years who are PrEP users.	Indirect contextual evidence	Sexualized drug use	Cannabis; club drugs (ketamine, ecstasy/MDMA, LSD, GHB, synthetic cathinones/bath salts); poppers; erectile stimulants; cocaine; solvents; crack cocaine. Recreational/sexual use.	Accessibility; acceptability; appropriateness. Difficulty in risk perception.	Supports the relevance of integrated sexual health services and risk perception among PrEP users.
SILVA, M.M.S. et al., 2025 [34], São Paulo—SP	To analyze the association between sexual orientation and gender identity with sexual practices, psychoactive substance use, and the occurrence of STIs among PrEP users.	Quantitative—Cross-sectional study	PrEP users.	Direct contextual evidence	Chemsex: “Intentional use of psychoactive substances associated with sexual practices to intensify and prolong sex.”	Alcohol; cannabis; poppers; cocaine; crack cocaine; club drugs; erectile stimulants; solvents. Use in sexual contexts. Sexual encounters.	Acceptability; accessibility; appropriateness. Stigma; institutional discrimination; racial, gender, and educational inequalities.	Reinforces the influence of intersectional inequities, stigma, and discrimination on healthcare access.

## Data Availability

No new data were created or analyzed in this study.

## Supplementary Materials

The following supporting information can be downloaded at: https://www.mdpi.com/article/10.3390/nursrep16070238/s1. Supplementary Material S1: Search strategies for all databases; Supplementary Material S2: PRISMA-ScR Checklist.

## Public Involvement Statement

No public involvement in any aspect of this research.

## Guidelines and Standards Statement

This manuscript was drafted in accordance with the Preferred Reporting Items for Systematic Reviews and Meta-Analyses extension for Scoping Reviews (PRISMA-ScR), as detailed in Supplementary Material S2.

## Abbreviations

The following abbreviations are used in this manuscript:

AIDS	Acquired Immunodeficiency Syndrome
CAPS	Psychosocial Care Centers (Centros de Atenção Psicossocial)
CTA	Testing and Counseling Centers (Centros de Testagem e Aconselhamento)
GBL	Gamma-Butyrolactone
GHB	Gamma-Hydroxybutyrate
HIV	Human Immunodeficiency Virus
MSM	Men Who Have Sex with Men
PEP	Post-Exposure Prophylaxis
PrEP	Pre-Exposure Prophylaxis
PCC	Population, Concept, and Context
PRISMA-ScR	Preferred Reporting Items for Systematic Reviews and Meta-Analyses Extension for Scoping Reviews
SAE	Specialized Care Services (Serviços de Atendimento Especializado)
SciELO	Scientific Electronic Library Online
SDU	Sexualized Drug Use
STIs	Sexually Transmitted Infections
SUS	Brazilian Unified Health System (Sistema Único de Saúde)
UBS	Basic Health Units (Unidades Básicas de Saúde)
