# Supplementary material for: Healthcare Access in Chemsex Contexts in Brazil: A Scoping Review and the VIP-Chemsex Model
Source: Nurs Rep. 2026 Jul 9;16(7):238. doi: 10.3390/nursrep16070238 (PMC13415104; doi:10.3390/nursrep16070238)
Supplement: Supplementary file 1 [file nursrep-16-00238-s001.zip › Supplementary Material S1 Search strategies for all databases (1).pdf]

Database/date	PCC	QUERY	RECORDS RETRIEVED
PubMed<br>November 5th, 2025	Participants<br><br>Concept<br><br>Context	Search: (("chemsex" OR "chemsex users" OR "chemical sex" OR "sexualized drug use" OR "sdu" OR "drug-facilitated sex" OR "sex under the influence of drugs" OR "high-risk sexual behavior" OR "party and play" OR "pnp" OR "high fun" OR "slamming" OR "recreational drug use" OR "club drug use" OR "illicit drugs" OR "polydrug use" OR "drug users" OR "substance-related disorders" OR "substance abuse, oral" OR "substance abuse, intravenous" OR "psychotropic drugs" OR "sexual behavior" OR "harm reduction" OR "addiction" OR "methamphetamine" OR "crystal meth" OR "ghb" OR "mephedrone" OR "ketamine" OR "mdma" OR "ecstasy" OR "cocaine" OR "poppers" OR "crack" OR "amphetamines" OR "lolo") AND ("health services accessibility" OR access* OR "availability of health services" OR "delivery of health care" OR "access to health services" OR "healthcare access" OR "health services access" OR "healthcare services" OR "public health services" OR "primary health care" OR "healthcare barriers" OR "health service utilization" OR "service utilization" OR "use of health services" OR "barriers and facilitators" OR "health disparities" OR "healthcare inequities" OR "healthcare inequalities" OR "health care needs" OR "healthcare needs" OR "unmet health needs" OR "unmet healthcare needs")) AND ("brazil" OR "brasil" OR "sus" OR "sistema unico de saude" OR "brazilian healthcare system" OR "acre" OR "alagoas" OR "amapa" OR "amazonas" OR "bahia" OR "ceara" OR "distrito federal" OR "espirito santo" OR "goias" OR "maranhao" OR "mato grosso" OR "mato grosso do sul" OR "minas gerais" OR "para" OR "paraiba" OR "parana" OR "pernambuco" OR "piaui" OR "rio de janeiro" OR "rio grande do norte" OR "rio grande do sul" OR "rondonia" OR "roraima" OR "santa catarina" OR "sao paulo" OR "sergipe" OR "tocantins") Filters: from 2014 - 2025<br><br>English, Portuguese, Spanish<br>Title, Abstract, Keyword	470

Database/date	PCC	QUERY	RECORDS RETRIEVED
Scopus<br>November 5th, 2025	Participants<br><br>Concept<br><br>Context	(TITLE-ABS-KEY ("chemsex" OR "chemsex users" OR "chemical sex" OR "sexualized drug use" OR "sdu" OR "drug-facilitated sex" OR "sex under the influence of drugs" OR "high-risk sexual behavior" OR "party and play" OR "pnp" OR "high fun" OR "slamming" OR "recreational drug use" OR "club drug use" OR "illicit drugs" OR "polydrug use" OR "drug users" OR "substance-related disorders" OR "substance abuse, oral" OR "substance abuse, intravenous" OR "psychotropic drugs" OR "sexual behavior" OR "harm reduction" OR "addiction" OR "methamphetamine" OR "crystal meth" OR "ghb" OR "mephedrone" OR "ketamine" OR "mdma" OR "ecstasy" OR "cocaine" OR **"powders"** OR "crack" OR "amphetamines" OR **"load"**) AND TITLE-ABS-KEY ("health services accessibility" OR access* OR "availability of health services" OR "delivery of health care" OR "access to health services" OR "healthcare access" OR "health services access" OR "healthcare services" OR "public health services" OR "primary health care" OR "healthcare barriers" OR "health service utilization" OR "service utilization" OR "use of health services" OR "barriers and facilitators" OR "health disparities" OR "healthcare inequities" OR "healthcare inequalities" OR "health care needs" OR "healthcare needs" OR "unmet health needs" OR "unmet healthcare needs") AND TITLE-ABS-KEY ("brazil" OR "brasil" OR "sus" OR "sistema unico de saude" OR "brazilian healthcare system" OR "acre" OR "alagoas" OR **"ampk"** OR "amazonas" OR "bahia" OR "ceara" OR "distrito federal" OR "espirito santo" OR "goias" OR "maranhao" OR "mato grosso" OR "mato grosso do sul" OR "minas gerais" OR "para" OR "paraiba" OR "parana" OR "pernambuco" OR "piaui" OR "rio de janeiro" OR "rio grande do norte" OR "rio grande do sul" OR "rondonia" OR "roraima" OR "santa catarina" OR "sao paulo" OR "sergipe" OR "tocantins"))<br><br>English, Portuguese, Spanish<br>Title, Abstract, Keyword	539

Database/date	PCC	QUERY	RECORDS RETRIEVED
Embase<br>November 5th, 2025	Participants<br><br>Concept<br><br>Context	("chemsex" OR "chemsex users" OR "chemical sex" OR "sexualized drug use" OR "sdu" OR "drug-facilitated sex" OR "sex under the influence of drugs" OR "high-risk sexual behavior" OR party and play OR "pnp" OR "high fun" OR "slamming" OR "recreational drug use" OR "club drug use" OR "illicit drugs" OR "polydrug use" OR "drug users" OR "substance-related disorders" OR "substance abuse, oral" OR "substance abuse, intravenous" OR "psychotropic drugs" OR "sexual behavior" OR "harm reduction" OR "addiction" OR "methamphetamine" OR "crystal meth" OR "ghb" OR "mephedrone" OR "ketamine" OR "mdma" OR "ecstasy" OR "cocaine" OR "poppers" OR "crack" OR "amphetamines" OR "lolo"):ti,ab,kw AND (("health services accessibility" OR access* OR "availability of health services" OR "delivery of health care" OR "access to health services" OR "healthcare access" OR "health services access" OR "healthcare services" OR "public health services" OR "primary health care" OR "healthcare barriers" OR "health service utilization" OR "service utilization" OR "use of health services" OR barriers and facilitators OR "health disparities" OR "healthcare inequities" OR "healthcare inequalities" OR "health care needs" OR "healthcare needs" OR "unmet health needs" OR "unmet healthcare needs"):ti,ab,kw) AND (("brazil" OR "brasil" OR "sus" OR "sistema unico de saude" OR "brazilian healthcare system" OR "acre" OR "alagoas" OR "amapa" OR "amazonas" OR "bahia" OR "ceara" OR "distrito federal" OR "espirito santo" OR "goias" OR "maranhao" OR "mato grosso" OR "mato grosso do sul" OR "minas gerais" OR "para" OR "paraiba" OR "parana" OR "pernambuco" OR "piaui" OR "rio de janeiro" OR "rio grande do norte" OR "rio grande do sul" OR "rondonia" OR "roraima" OR "santa catarina" OR "sao paulo" OR "sergipe" OR "tocantins"):ti,ab,kw) AND [2014-2025]/py<br><br>English, Portuguese, Spanish<br>Title, Abstract, Keyword	777

Database/date	PCC	QUERY	RECORDS RETRIEVED
SciELO<br>November 5th, 2025	Participants<br><br>Concept<br><br>Context	(("chemsex" OR "chemsex users" OR "chemical sex" OR "sexualized drug use" OR "sdu" OR "drug-facilitated sex" OR "sex under the influence of drugs" OR "high-risk sexual behavior" OR "party and play" OR "pnp" OR "high fun" OR "slamming" OR "recreational drug use" OR "club drug use" OR "illicit drugs" OR "polydrug use" OR "drug users" OR "substance-related disorders" OR "substance abuse, oral" OR "substance abuse, intravenous" OR "psychotropic drugs" OR "sexual behavior" OR "harm reduction" OR "addiction" OR "methamphetamine" OR "crystal meth" OR "ghb" OR "mephedrone" OR "ketamine" OR "mdma" OR "ecstasy" OR "cocaine" OR "poppers" OR "crack" OR "amphetamines" OR "lolo") AND ("health services accessibility" OR access* OR "availability of health services" OR "delivery of health care" OR "access to health services" OR "healthcare access" OR "health services access" OR "healthcare services" OR "public health services" OR "primary health care" OR "healthcare barriers" OR "health service utilization" OR "service utilization" OR "use of health services" OR "barriers and facilitators" OR "health disparities" OR "healthcare inequities" OR "healthcare inequalities" OR "health care needs" OR "healthcare needs" OR "unmet health needs" OR "unmet healthcare needs")) AND ("brazil" OR "brasil" OR "sus" OR "sistema unico de saude" OR "brazilian healthcare system" OR "acre" OR "alagoas" OR "amapa" OR "amazonas" OR "bahia" OR "ceara" OR "distrito federal" OR "espirito santo" OR "goias" OR "maranhao" OR "mato grosso" OR "mato grosso do sul" OR "minas gerais" OR "para" OR "paraiba" OR "parana" OR "pernambuco" OR "piaui" OR "rio de janeiro" OR "rio grande do norte" OR "rio grande do sul" OR "rondonia" OR "roraima" OR "santa catarina" OR "sao paulo" OR "sergipe" OR "tocantins"))<br>Filters: from 2014 - 2025<br><br>English, Portuguese, Spanish<br>Title, Abstract, Keyword	1.374

Database/date	PCC	QUERY	RECORDS RETRIEVED
LILACS<br>November 5th, 2025	Participants<br>Concept<br>Context	("chemsex" OR "chemsex users" OR "chemical sex" OR "sexualized drug use" OR "sdu" OR "drug-facilitated sex" OR "sex under the influence of drugs" OR "high-risk sexual behavior" OR "party and play" OR "pnp" OR "high fun" OR "slamming" OR "recreational drug use" OR "club drug use" OR "illicit drugs" OR "polydrug use" OR "drug users" OR "substance-related disorders" OR "substance abuse, oral" OR "substance abuse, intravenous" OR "psychotropic drugs" OR "sexual behavior" OR "harm reduction" OR "addiction" OR "methamphetamine" OR "crystal meth" OR "ghb" OR "mephedrone" OR "ketamine" OR "mdma" OR "ecstasy" OR "cocaine" OR "poppers" OR "crack" OR "amphetamines" OR "lolo") AND ("health services accessibility" OR access* OR "availability of health services" OR "delivery of health care" OR "access to health services" OR "healthcare access" OR "health services access" OR "healthcare services" OR "public health services" OR "primary health care" OR "healthcare barriers" OR "health service utilization" OR "service utilization" OR "use of health services" OR "barriers and facilitators" OR "health disparities" OR "healthcare inequities" OR "healthcare inequalities" OR "health care needs" OR "healthcare needs" OR "unmet health needs" OR "unmet healthcare needs") AND ("brazil" OR "brasil" OR "sus" OR "sistema unico de saude" OR "brazilian healthcare system" OR "acre" OR "alagoas" OR "amapa" OR "amazonas" OR "bahia" OR "ceara" OR "distrito federal" OR "espirito santo" OR "goias" OR "maranhao" OR "mato grosso" OR "mato grosso do sul" OR "minas gerais" OR "para" OR "paraiba" OR "parana" OR "pernambuco" OR "piaui" OR "rio de janeiro" OR "rio grande do norte" OR "rio grande do sul" OR "rondonia" OR "roraima" OR "santa catarina" OR "sao paulo" OR "sergipe" OR "tocantins") AND instance:"lilacsplus"<br><br>English, Portuguese, Spanish<br>Title, Abstract, Subject title	1.478

Database/date	PCC	QUERY	RECORDS RETRIEVED
PsycINFO<br>November 5th, 2025	Participants<br><br>Concept<br><br>Context	Results for **Any Field**: "chemsex" *OR* **Any Field**: "chemsex users" *OR* **Any Field**: "chemical sex" *OR* **Any Field**: "sexualized drug use" *OR* **Any Field**: "sdu" *OR* **Any Field**: "drug-facilitated sex" *OR* **Any Field**: "sex under the influence of drugs" *OR* **Any Field**: "high-risk sexual behavior" *OR* **Any Field**: "party and play" *OR* **Any Field**: "pnp" *OR* **Any Field**: "high fun" *OR* **Any Field**: "slamming" *OR* **Any Field**: "recreational drug use" *OR* **Any Field**: "club drug use" *OR* **Any Field**: "illicit drugs" *OR* **Any Field**: "polydrug use" *OR* **Any Field**: "drug users" *OR* **Any Field**: "substance-related disorders" *OR* **Any Field**: "substance abuse, oral" *OR* **Any Field**: "substance abuse, intravenous" *OR* **Any Field**: "psychotropic drugs" *OR* **Any Field**: "sexual behavior" *OR* **Any Field**: "harm reduction" *OR* **Any Field**: "addiction" *OR* **Any Field**: "methamphetamine" *OR* **Any Field**: "crystal meth" *OR* **Any Field**: "ghb" *OR* **Any Field**: "mephedrone" *OR* **Any Field**: "ketamine" *OR* **Any Field**: "mdma" *OR* **Any Field**: "ecstasy" *OR* **Any Field**: "cocaine" *OR* **Any Field**: "poppers" *OR* **Any Field**: "crack" *OR* **Any Field**: "amphetamines" *OR* **Any Field**: "lolo" *AND* **Any Field**: "health services accessibility" *OR* **Any Field**: access* *OR* **Any Field**: "availability of health services" *OR* **Any Field**: "delivery of health care" *OR* **Any Field**: "access to health services" *OR* **Any Field**: "healthcare access" *OR* **Any Field**: "health services access" *OR* **Any Field**: "healthcare services" *OR* **Any Field**: "public health services" *OR* **Any Field**: "primary health care" *OR* **Any Field**: "healthcare barriers" *OR* **Any Field**: "health service utilization" *OR* **Any Field**: "service utilization" *OR* **Any Field**: "use of health services" *OR* **Any Field**: "barriers and facilitators" *OR* **Any Field**: "health disparities" *OR* **Any Field**: "healthcare inequities" *OR* **Any Field**: "healthcare inequalities" *OR* **Any Field**: "health care needs" *OR* **Any Field**: "healthcare needs" *OR* **Any Field**: "unmet health needs" *OR*<br><br>English, Portuguese, Spanish<br>Any Field	673
